# Impaired Skeletal Muscle Branched-Chain Amino Acids Catabolism Contributes to Their Increased Circulating Levels in a Non-Obese Insulin-Resistant Fructose-Fed Rat Model

**DOI:** 10.3390/nu11020355

**Published:** 2019-02-08

**Authors:** Jérémie David, Dominique Dardevet, Laurent Mosoni, Isabelle Savary-Auzeloux, Sergio Polakof

**Affiliations:** Université Clermont Auvergne, INRA, Unité de Nutrition Humaine, UMR1019, F-63000 CLERMONT-FERRAND, France; jeremie.david@inra.fr (J.D.); dominique.dardevet@inra.fr (D.D.); laurent.mosoni@inra.fr (L.M.); isabelle.savary-auzeloux@inra.fr (I.S.-A.)

**Keywords:** fructose, insulin resistance, skeletal muscle, branched-chain amino acids, liver, obesity

## Abstract

Elevated plasma branched-chain amino acids (BCAA) levels are often observed in obese insulin-resistant (IR) subjects and laboratory animals. A reduced capacity of the adipose tissues (AT) to catabolize BCAA has been proposed as an explanation, but it seems restricted to obesity models of genetically modified or high fat–fed rodents. We aimed to determine if plasma BCAA levels were increased in a model of IR without obesity and to explore the underlying mechanisms. Rats were fed with a standard diet, containing either starch or fructose. BCAA levels, body weight and composition were recorded before and after 5, 12, 30, or 45 days of feeding. Elevated blood BCAA levels were observed in our IR model with unaltered body weight and composition. No changes were observed in the liver or the AT, but instead an impaired capacity of the skeletal muscle to catabolize BCAA was observed, including reduced capacity for transamination and oxidative deamination. Although the elevated blood BCAA levels in the fructose-fed rat seem to be a common feature of the IR phenotype observed in obese subjects and high fat–fed animals, the mechanisms involved in such a metabolic phenomenon are different, likely involving the skeletal muscle BCAA metabolism.

## 1. Introduction

The place of amino acids (namely branched chain amino acids, BCAA) has been recently questioned in the metabolic dysfunctions and plasma metabolites alterations associated with insulin resistance (IR) occurrence [1]. Indeed, an increased level of plasma BCAA was often observed in IR individuals and shown to be altered during or even before the IR and diabetes installation [1,2,3]. To date, the underlying mechanisms explaining these increased BCAA levels is still under debate [4,5] and it remains controversial if the increased level of BCAA should be considered as causal of IR or a consequence of the IR development [5]. Among the mechanisms involved in this phenotype, a decreased capacity of the whole body to catabolize BCAA has been recently investigated, and a particular reduction of the BCAA catabolic enzymes in the adipose tissue has been reported in both animal model and humans [5,6,7]. It is however unclear whether increased BCAA results from altered adipose tissue metabolism associated with obesity or if it originally results from the IR development, which will lead to obesity and dysfunction of BCAA metabolism in the adipose cells [1,8]. Other hypotheses explaining the increased BCAA levels profile include the permanent stimulation of the mTOR (mammalian target of rapamycin) signaling pathway by circulating BCAA, which would further induce a degradation of the IRS proteins, leading to IR [9,10]. Despite the possible role of the adipose tissue on this altered BCAA phenotype, the contribution of other tissues to this perturbed BCAA metabolism and/or their metabolites production has been less studied in such situations. This is the case of the liver which is poorly involved in BCAA transamination but is a major actor in BCKA (branched-chain keto acids) catabolism [11,12,13]. However, it has been shown that its role on BCAA metabolism could change according to the pathophysiological condition. Thus, in obese and IR high fat–fed rats, the liver oxidative deamination capacity for BCKA could be enhanced to compensate the adipose tissue impaired metabolism in order to maintain a whole-body BCAA homeostasis [13]. Similarly, it has been shown that the adipose tissue is not the only organ in which BCAA catabolism could be impaired in the context of metabolic disorders. Accordingly, it has been shown in patients with different degrees of obesity and insulin sensitivity that a defect on BCAA oxidation in the skeletal muscle could contribute to impaired lipid metabolism and IR [14]. Further, a recent study also evidenced that the altered BCAA levels could be the consequence of the IR based on alteration in several organs, including liver, skeletal muscle, and adipose tissue [15].

Considering that an alteration of the catabolic capacities of the adipose tissues may not be the only explanation for the increased BCAA plasma level observed in IR development [1], the aim of the present work was to follow kinetically BCAA plasma levels in an experimental model of IR onset that develops without significant increase in body weight or changes in body composition. This model of fructose-induced IR has been chosen as it impacts primarily the liver and is known to rapidly induce the generation of hepatic steatosis and skeletal muscle IR and via a drastic alteration of glucose and lipid metabolism [16,17,18]. Such a model may help to decipher if BCAA levels can also be modified when animals are not obese and if the metabolism of other organs, like the liver or the skeletal muscle, is already affected.

## 2. Materials and Methods

### 2.1. Animals

Eight-week-old Sprague-Dawley male rats (Charles River, L’Arbresle Cedex, France) were housed individually and kept in a controlled environment (temperature maintained at 22 °C; 12:12 light: dark cycle).

### 2.2. Experimental Procedure

After one week adaptation, animals were divided into two groups and fed experimental diets (based on AIN 93G diet), containing as source of carbohydrates (~63%), either starch (control group, *n* = 40) or fructose (fructose group, *n* = 40), during 45 days. Rats had free access to fresh tap water. The control group was pair-fed with respect to the fructose group in order to match the daily food intake. The animals were handled according to the recommendations of the Regional Ethics Committee (agreement number CE-2811). All procedures were in accordance with the guidelines formulated by the European Community for the use of experimental animals (L358-86/609/EEC, Council Directive, 1986).

Before (d0) and after 5, 12, 30 or 45 days of feeding blood was withdrawn at the postabsorptive (overnight fasting) and postprandial (2 h after the meal) state from the tail vein. To avoid the effect of stress by sampling too often (d0, d5, d12, d30 and d45), eight different animals per diet (control or fructose) were randomly assigned to the five groups and sampled only at one time. Body weight and body composition (EchoMRITM-700; Texas, USA) were evaluated at the same days than the blood sampling. Those animals assigned to the d45 sampling were also euthanized under pentobarbital anesthesia in the post-absorptive state (overnight fasting). The liver, the subcutaneous and epididymal adipose tissues, and the gastrocnemius muscle were rapidly removed, weighed, freeze-clamped into liquid nitrogen, and stored at −80 °C for further analyses. Two days before the end of the trial, the eight rats per group sampled at d0 were further subjected to an oral glucose tolerance test, in which they received orally 1 g of glucose/kg of body weight. Blood glucose levels were then assessed before (0 min) and 15, 30, 60, 90, and 120 min after the test using a glucometer (Accu-chek, Roche). The areas under the curves (AUC) of the glucose excursion was calculated by the trapezoid method.

### 2.3. Analytical Procedures

Plasma glucose and triglycerides concentrations were enzymatically measured using commercial kits (BioMerieux, Marcy-l’Etoile, France). Plasma insulin levels were assessed using a commercial ELISA kit (Millipore, Burlington, MA, USA). Glucose homeostasis was further estimated using the homeostasis model assessment-estimated insulin resistance using the HOMA2-IR model (from www.OCDEM.ox.ac.uk) as in Levy et al. [19]. Total BCAA levels were determined enzymatically on plasma samples in the presence of leucine dehydrogenase (2 U/sample) and NAD^+^ (4 mM). The amount of BCAA was calculated based on the NADH formation using a standard leucine curve as in [20,21]. BCAT2 (branched-chain-amino-acid aminotransferase) enzyme activity was determined as in [21,22] by following the NADH consumption at 340 nm in the presence of Tris pH 8.5, NADH, pyridoxal 5-phosphate, and excess of glutamate, aspartate, malic dehydrogenase, and aspartate aminotransferase. The substrate (ketoisocaproic acid, 150 μM) was omitted in the blank. Protein was determined by the BCA method. Hepatic triglyceride levels were extracted and analyzed by the Folch method [23] with some modifications [24].

For Western blot analyses, total protein lysates (20 μg for liver and adipose tissues, and 40 μg for muscle) were subjected to SDS-PAGE, electrotransferred on a PVDF membrane, and probed with the indicated antibodies: total BCKDH (branched-chain α-ketoacid dehydrogenase complex) and phosphorylated BCKDH-α at serine 293 (Abcam); total S6 (ribosomal protein S6, phosphorylated) and phosphorylated S6 at Ser240/244 antibodies (Cell Signaling Technology, Ozyme, St Quentin-en-Yvelines, France). Bands were visualized by infrared fluorescence using the Odyssey imaging system. Phosphorylated proteins were corrected for the respective total proteins. The signal of each protein was further normalized by the total protein content of the gel (Stain-Free Imaging Technology, Bio-Rad) [25].

Total RNA was extracted from livers and gastrocnemius muscles using RNEasy Mini Kit^®^ (Qiagen, Hilden, Germany) and mRNA levels were determined by RT-PCR. cDNA was generated from 1 μg RNA using High Capacity cDNA Reverse Transcription Kit (Life Technologies, France). Real-time PCR was performed in the CFX96 Touch™ Real-Time PCR Detection System (BIO-RAD, Hercules, CA, USA) as in [26]. Primers were designed so that they are overlapping an intron (Primer3 software; Whitehead Institute for Biomedical Research/MIT Center, Cambridge, MA, USA) using known sequences in nucleotide databases and are available upon request.

The differences in BCAA levels during the time-course experiment and glucose levels in the oral glucose tolerance test were analyzed using a two-way ANOVA test, followed by post-hoc Holm-Sidak comparison (SigmaPlot 12, Systat Software, San Jose, CA, USA). The differences between groups in the parameters evaluated at the end of the trial were performed using a t-test analysis. The *p*-value significance threshold was set at *p* < 0.05.

## 3. Results

**Figure 1:**
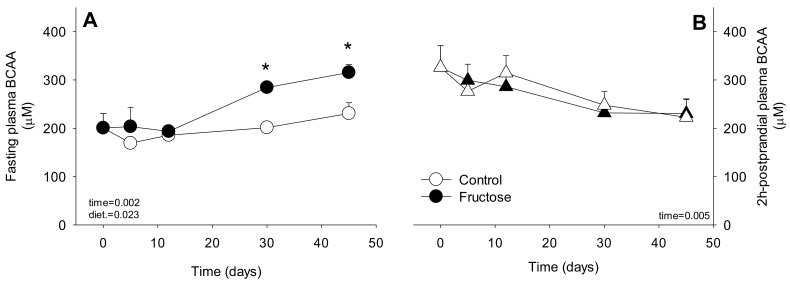
Plasma branched-chain amino acids (BCAA) levels (μM) at the fasting (overnight) and fed (2 h after the last meal) in rats fed a control or high-fructose diet for 45 days. Data is presented as mean ± SEM (*n* = 8). * represent significant (*p* < 0.05) differences between groups at a given time.

The plasma BCAA levels during the time-course fructose feeding in both, the fasting and postprandial states are shown in Figure 1. While BCAA fasting levels (Figure 1A) increased gradually in the fructose-fed animals (significantly at d30 and d45), the post prandial levels of BCAA levels measured 2 h after the meal remained identical in both groups (Figure 1B).

Blood glucose and insulin levels were also assessed at the fasting state (Table 1). All the parameters showed increased levels with time, but only glucose and HOMA-IR were affected by the diet. Blood glucose levels were significantly different between the control and the fructose groups from day 12 up to the end of the trial. For insulin levels and HOMA-IR, the differences among the diets were only significant at d45.

**Figure 2:**
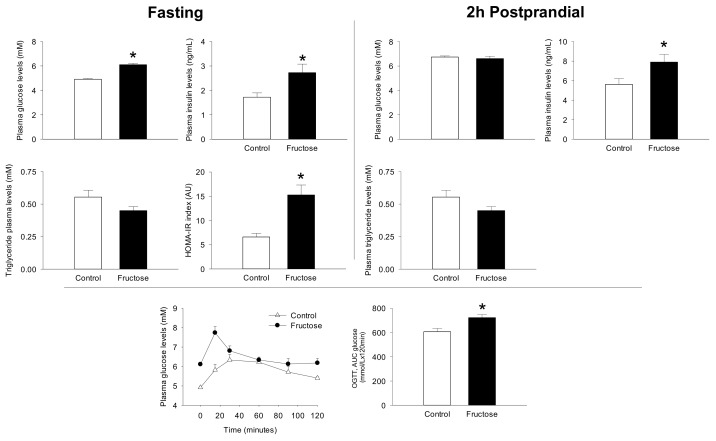
*Upper panel:* plasma glucose, insulin and triglyceride levels as well as HOMA-IR index at the fasting and postprandial state (2 h after the last meal) on rats after 45 days of feeding a control or high-fructose diet. *Lower panel:* plasma glucose levels and area under the curve (AUC) during oral glucose tolerance test (OGTT). Data is presented as mean ± SEM (*n* = 8). * represent significant (*p* < 0.05) differences between groups. ND, not detected.

Based on the well-established relationship between fructose feeding and IR on rodent models [18], and between IR and elevated BCAA levels [27] we therefore characterized the glucose homeostasis status of the animals from the present study (Figure 2). Thus, by the end of the trial fructose-fed rats showed significant fasting hyperglycemia (+24%) and hyperinsulinemia (+58%) when compared to the control group, both symptoms of impaired glucose homeostasis. In contrast, at the postprandial level, fructose-fed rats only displayed significant hyperinsulinemia (+41%) and normal blood glucose levels in comparison to the starch-fed animals. Although, no significant differences were observed in blood triglyceride levels among the groups irrespectively of the nutritional conditions (fed or fasted), we did observe a significant increase in the hepatic triglyceride content (control, 52.28 µg/g ± 4.33 vs. fructose, 97.18 ± 15.09 µg/g). Taking into account the perturbed blood glucose and insulin levels, we further evaluated glucose homeostasis by calculating the HOMA-IR index. Fructose fed rats had a two-fold increase in HOMA-IR index when compared to the control group, which is a major symptom of IR. Further, the fructose-fed animals were also glucose intolerant, as shown by the perturbed blood glucose curve after the OGTT and the significantly exacerbated AUC (+20%).

**Figure 3:**
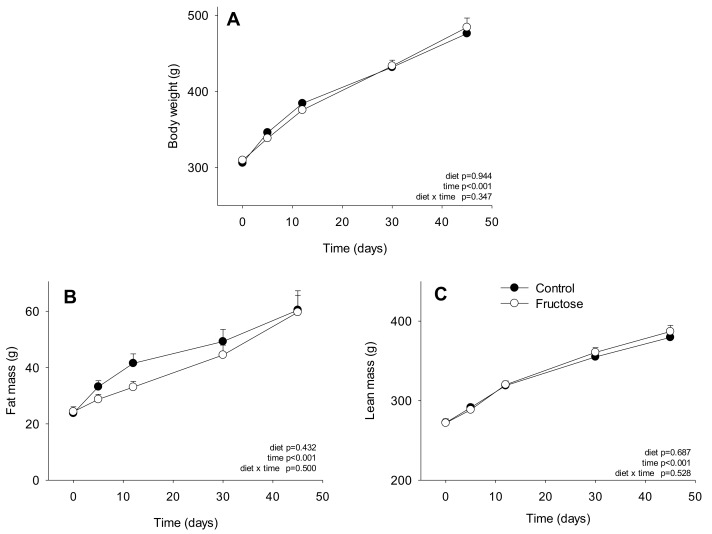
Body weight (**A**), fat mass (**B**), and lean mass (**C**) of rats fed on a control or high-fructose diet for 45 days. Data is presented as mean ± SEM (*n* = 8). Data is expressed in grams (g) and was analyzed using two-way ANOVA test.

Taking into account the major role of the adipose tissue in BCAA metabolism described in the literature [5,6,7], we recorded during the whole trial the body weight and the body composition using a non-invasive method (Figure 3).

Body weight, fat mass, and lean mass increased significantly with the time, but none of these parameters was affected by the IR induced by the fructose feeding (*p*-value for diet was 0.94, 0.43, and 0.69 respectively).

**Figure 4:**
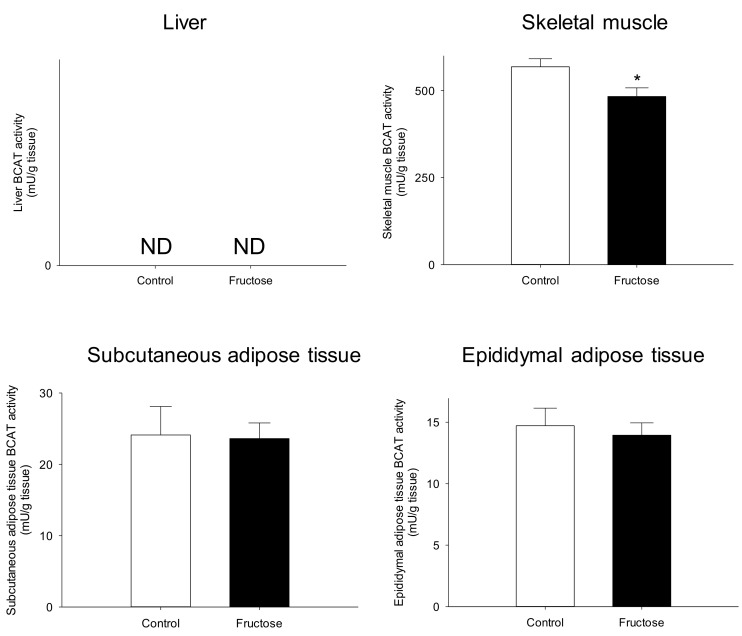
Branched chain aminotransferase 2 (BCAT2) activity in the liver, skeletal muscle, subcutaneous adipose tissue, and epididymal adipose tissue of rats fed on a control or high-fructose diet for 45 days. Data is presented as mean ± SEM (*n* = 8). * represent significant (*p* < 0 .05) differences between groups. ND, not detected.

**Figure 5:**
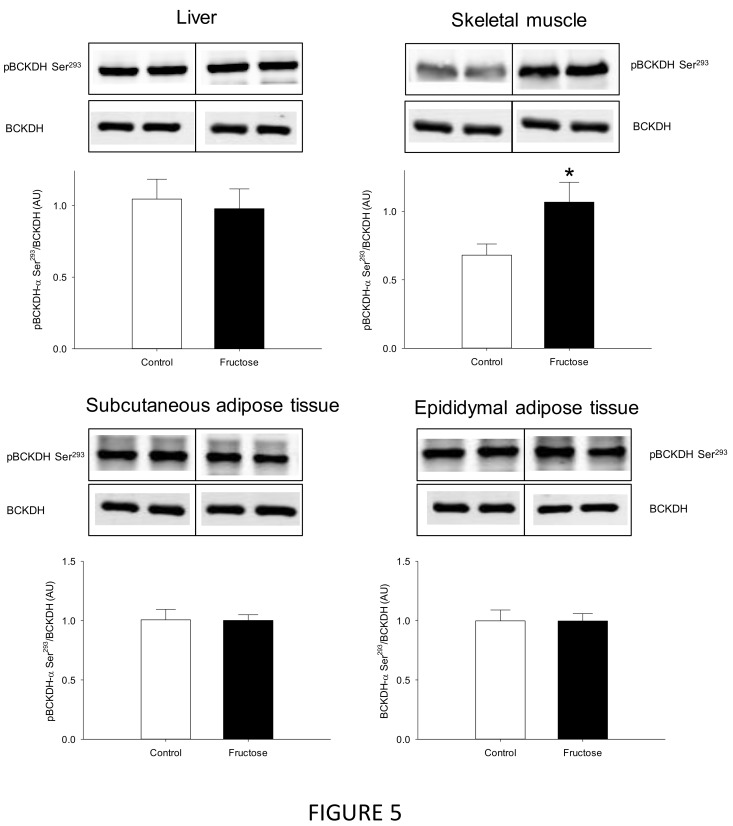
Representative Western blot analyses of branched-chain alpha-keto acid dehydrogenase (BCKDH) complex phosphorylation status (Ser^293^) in the liver, skeletal muscle subcutaneous adipose tissue, and epididymal adipose tissue of rats fed a control or high-fructose diet during 45 days. Data is presented as mean ± SEM (*n* = 8). * represent significant (*p* < 0.05) differences between groups.

The next step was to explore the capacity of the liver, adipose tissues and the skeletal muscle to catabolize BCAA, which has been done by assessing the transamination (BCAT activity) and oxidative deamination (phosphorylation status of BCKDH) potentials (Figure 4 and Figure 5, respectively). While BCAT activity was not detected in the liver homogenates, its activity was reduced in the skeletal muscle (–15%) and remained unchanged in both adipose tissues (subcutaneous and epididymal). The BCKDH phosphorylation status remained also unaltered in the liver and the adipose tissues, but it was increased by 43% in the skeletal muscle.

**Figure 6:**
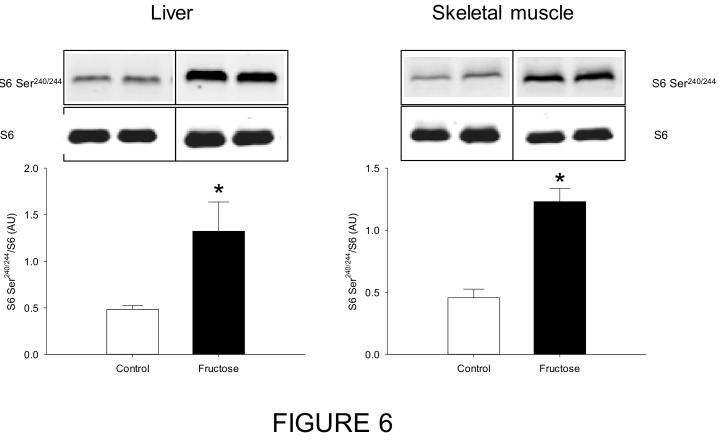
Representative Western blot analyses of S6 protein complex phosphorylation status (S6 Ser^240/244^/S6) in the liver and skeletal muscle of rats fed a control or high-fructose diet during 45 days. Data is presented as mean ± SEM (*n* = 8). * represent significant (*p* < 0.05) differences between groups.

We also intended to understand the mechanisms behind these changes by studying the mTOR signaling pathway and the expression of several proteins and transcription factors involved in the potential regulation of BCAA metabolism in the liver and the skeletal muscle. The phosphorylation status of the mTOR signaling pathway maker, S6, was strongly up-regulated by about 2.7-fold in both the muscle and the liver (Figure 6).

Regarding the mRNA levels, only *mlxipl* (coding for ChREBP, carbohydrate-responsive element-binding protein) expression was affected at the hepatic levels, with a 1.3-fold increase in the fructose-fed rats. In the skeletal muscle, this transcription factor was instead reduced by 1.5-fold. Further, both *ppargc1a* and *sirt1* (sirtuin 1) were also down-regulated (about 1.5-fold) in the IR animals in comparison to the control group (Table 2). 

## 4. Discussion

In the present study we showed for the first time that chronic fructose consumption for 45 days resulted in increased fasting BCAA levels. These increased BCAA levels were associated with the installation of a whole-body glucose intolerance and IR. The originality of the present model of IR development was that the most important tissue targeted by the fructose consumption is the liver, but instead, we showed that altered skeletal muscle BCAA metabolism seems to be responsible for the elevated circulating BCAA levels. Further, the lack of changes in body composition, particularly fat mass strongly suggests a minor role of the adipose tissue on BCAA elevation, at least in this particular model.

Over the last years, IR phenotype has been demonstrated to be associated with higher circulating levels of plasma fasting BCAA [10]. Further, in longitudinal studies in humans, these BCAA levels have been shown as able to predict the appearance of IR/T2DM phenotypes [28], suggesting a causal role of BCAA in the development of IR. In this sense, it is worth mentioning that most of these results were obtained in humans or animal models in which IR was the direct consequence of a particular obese genetic background in rodents (Zucker rats, *db/db* mice) [7] or nutritionally induced by the consumption of high-fat diet known to increase fat mass and/obesity rapidly in both rodent and swine models [21,29]. Here, we presented elevated BCAA levels in a model of nutritionally induced IR: the fructose-fed rat which after 45 days of feeding was not associated with increased body weight, fat mass or high energy intake. Fasting BCAA levels have been measured in a time-course manner and increased gradually during the 45 days of fructose feeding. Our results show that BCAA levels increase very early, at least 30 days after fructose feeding, suggesting that they could precede the IR installation, the onset of which usually needs more than one month in this particular model [30,31,32]. Further, the fructose-fed rats exhibited a higher suppression of the increase in BCAA levels following the meal, most likely due to the significant higher postprandial hyperinsulinemia that would facilitate the amino acids uptake by peripheral tissues [33].

Very few data are available concerning BCAA levels in an IR model induced by fructose: most of the studies dealing with fructose impact on AA (and BCAA) metabolism have focused on the nitrogen sparing effect of fructose supplementation [34] and not on a pathophysiological situation (such as fructose-induced IR). More recently, two studies presented plasma AA levels in fructose-fed rats for a longer period of time (eight weeks), which led to the development of NAFLD [31], one of them showing concomitant IR [32], without reporting any significant effect on BCAA levels by the end of the trial. Finally, Borzinick et al. [35] observed elevated valine levels in diabetic macaques drinking a fructose-enriched solution (20%) three times a week, but the fact that they also received a high fat diet makes the comparison with our study difficult. Finally, a more recent study showed that a BCAA and lipid metabolisms could be interconnected at the hepatic level following a single fructose meal [36].

Among the potential mechanisms able to alter the circulating levels of BCAA, their utilization and oxidation by the different targeted tissues should be considered. In several models of obesity and IR, it has been shown that the increased BCAA levels in blood were the consequence of a reduced capacity of the adipose tissue (importantly developed) to transaminate BCAA and oxidize their ketoacids [5,6]. In the present study, after 45 days of fructose feeding, IR animals did not display any change in body weight. More interestingly, fat mass resulted also unaltered. This constitute a major difference with the other rodent and swine models described in the literature, such as those fed on high fat diets [21], Zucker rats or *db/db* mice [7]. The fact that the fat mass evolved similarly between the control and fructose-fed rats could suggest that the adipose tissue in this particular model could have a minor role on BCAA homeostasis. This was further supported by the fact that the transamination potential (BCAT activity) and BCKDH phosphorylation status in both adipose tissues (epididymal and subcutaneous) were no modified in the fructose-fed rats when compared to the control group. Overall, our data support the idea that the potential of the adipose tissues to catabolize BCAA was overall low (even by unit of tissue mass) and could not be responsible of BCAA accumulation in the circulation.

As explained above, as BCAA can be potentially transaminated by several tissues, their whole-body homeostasis is highly dependent on the different organs cross-talk. Thus, previous studies have shown that in diet-induced obesity models liver BCKDH activity could be actually increased and compensate the reduced activity observed in the adipose tissues [13]. We therefore, explored this possibility in our fructose-fed rats, for which BCAT activity was not detected, and the BCKDH phosphorylation status resulted unchanged as for the adipose tissues. In contrast to this situation, we did find a reduced BCAT activity in the skeletal muscle of the IR fructose-fed rats. Given that this enzyme catalyzes a reversible reaction, we further explored the oxidative deamination potential. As expected, we observed that the phosphorylation status of the BCKDH complex was enhanced (then the activity inhibited) in the IR animals, which would result in a reduced capacity to oxidize ketoacids. A few studies also explored the oxidative potential of the skeletal muscle in genetically modified rodents, but the results are contradictory: while it remained unaffected in some studies [5] it was strongly reduced (−60%) in other [37]. A more recent study in humans (IR and diabetic) and rodents reported an impaired transport of BCAA into the muscle, and a reduction in BCAA oxidation in this tissue, which could explain the increase in BCAA levels [14]. Concerning the mechanism responsible for such impaired catabolic capacity at the muscle level, very little information is available from the literature. Thus, PGC1-α (peroxisome proliferator-activated receptor gamma coactivator 1-alpha) overexpression in transgenic mice resulted in increased BCAT and BCKDH expression in the skeletal muscle [38], suggesting a regulatory function on BCAA metabolism for this transcription factor. Other studies pointed to a mitochondrial dysfunction to explain the reduced ability of the skeletal muscle to oxidize BCAA, particularly at the dietary IR induction [1]. Finally, a recent study also demonstrated the hepatic BDK (branched chain ketoacid dehydrogenase kinase) and PPM1K (protein phosphatase 1K) tandem (responsible for BCKDH phosphorylation-dephosphorylation) and several genes involved in lipogenesis were regulated by the same transcription factor: ChREBP following a single fructose meal [36]. In our study we explored several of these possibilities in both, the liver and skeletal muscle, the most active organs concerning BCAA catabolism. Our results show tissue-dependent regulation of ChREBP mRNA levels in the fructose-fed animals, with increased levels in the liver and reduced in the skeletal muscle. At the hepatic level our results disagree with the regulatory function suggested by White et al. [36], but they are in line with several studies showing an activation of ChREBP in the context of a high-fructose diet [39]. These discrepancies could be due to the animal model but also to the time exposure to the diet (acute vs. chronic). In our case, given the important lipid accumulation observed in the liver, the enhanced ChREBP expression would be likely related to an adaptative mechanism aiming at protecting the liver from a further progression towards steatosis in the context of a long-term fructose feeding [40]. Concerning the skeletal muscle, the reduced ChREBP mRNA levels observed in our IR rats seem also coherent with the reduced glucose uptake induced also in rats for which ChREBP was reduced by antisense oligonucleotide injections [41]. Finally, we also observed at the skeletal muscle level a significant reduction of two markers of mitochondrial function, PGC-1α and SIRT1, which could suggest an IR-related mitochondrial number or dysfunction, likely responsible for the reduced oxidative capacity of the muscle to catabolize not only BCAA but also lipids [14].

In order to fully characterize the model, we also explored the mTOR signaling pathway, which has been suggested to be related to the IR observed in this kind of phenotype. We observed that the mTOR signaling pathway (S6 phosphorylation) was enhanced in the liver and skeletal muscle of fructose-fed rats. Today it is well known that persistent nutrient signaling like elevated BCAA levels might cause IR due to a continuous activation of the mTORC1 signaling pathway [9]. Several studies have shown that supplementation of BCAA, or infusion of a complete mixture of amino acids, activates the mTOR/S6K1 pathway leading to inhibitory serine phosphorylation of IRS1 [10]. Further, Zucker rats fed a BCAA restricted diet showed skeletal muscle improved insulin sensitivity [42]. We therefore may hypothesize that in the fructose-fed rats an impaired nutrient signaling induced by the elevated blood BCAA levels could operate in both liver and muscle and participate to the further IR onset. Given the controversial role of mTOR on IR linked to BCAA [8], other mechanisms should be further explored, such as the accumulation of toxic BCAA-derived metabolites.

As a whole, we showed for the first time that the installation of whole body fructose-induced IR was associated with increased fasting BCAA levels. Further, in the fructose-fed rats BCAA increase took place in the absence of significant changes in body weight and fat mass, a major difference with other models (high fat, Zucker, *db/db*), that suggests a minor role of the adipose tissue on BCAA homeostasis, at least in this particular model (IR but not obese). Our results also showed that particularly at the skeletal muscle level a reduction in both transamination and oxidative deamination potentials operates, suggesting that these features may participate to the elevated blood BCAA profile. The phenotype of these animals was also characterized by a reduction in several mitochondrial function markers (skeletal muscle) and an enhanced mTOR signaling pathway (liver and skeletal muscle) in the IR animals, opening the door to the exploration of further mechanisms linking high circulating BCAA levels and IR. Finally, the fact that after 45 days of fructose feeding rats did develop IR but not obesity makes this model a very interesting tool to further elucidate the specific role of the different tissues (adipose, liver, muscle…) and the relationship between obesity and IR in the context of elevated BCAA levels.

## Figures and Tables

**Table 1:** Fasting plasma glucose and insulin levels and HOMA2-IR (homeostasis model assessment-estimated insulin resistance) index of rats fed on a control or high-fructose diet for 45 days. Data is presented as mean ± SEM (*n* = 8). Data was analyzed using two-way ANOVA test. *, significantly different from the control condition at a given time.

		0d	5d	12d	30d	45d	*p*-Value
Glucose (mM)	Con	4.20 ± 0.14	4.43 ± 0.18	4.22 ± 0.30	4.87 ± 0.13	5.28 ± 0.23	Diet <0.001 Time <0.001 Diet × Time 0.214
	Fru	4.55 ± 0.33	4.49 ± 0.20	4.86 ± 0.26*	5.23 ± 0.10*	6.10 ± 0.11 *	
Insulin (ng/mL)	Con	0.77 ± 0.18	1.06 ± 0.13	1.46 ± 0.26	1.02 ± 0.18	1.72 ± 0.22	Diet 0.100 Time 0.004 Diet × Time 0.164
	Fru	1.09 ± 0.31	0.91 ± 0.15	1.34 ± 0.32	1.40 ± 0.37	2.58 ± 0.26*	
HOMA2-IR	Con	3.18 ± 0.75	4.60 ± 0.54	6.27 ± 1.26	5.67 ± 0.85	6.83 ± 0.49	Diet 0.010 Time <0.001 Diet × Time 0.042
	Fru	5.31 ± 1.92	4.04 ± 0.75	6.49 ± 1.57	7.75 ± 2.52	15.66 ± 1.90 *	

**Table 2:** mRNA levels of protein and transcription factors involved in BCAA metabolism regulation on the liver and skeletal muscle of rats fed on a regular or high-fructose diet for 45 days.

	Liver	
	*Control*	*Fructose*
*ppargc1a*	1.24 ± 0.38	1.10 ± 0.16
*pparg*	0.84 ± 0.05	0.96 ± 0.17
*mlxipl*	0.99 ± 0.09	1.27 ± 0.11 *
	**Skeletal muscle**	
*ppargc1a*	1.05 ± 0.20	0.76 ± 0.06 *
*sirt1*	1.06 ± 0.23	0.72 ± 0.07 *
*mlxipl*	1.17 ± 0.42	0.80 ± 0.15 *

*ppargc1a*, peroxisome Proliferator-Activated Receptor Gamma, Coactivator 1 Alpha; *pparg,* Peroxisome Proliferator Activated Receptor Alpha; *mlxipl*, Carbohydrate-Responsive Element-Binding Protein; *sirt1,* Sirtuin 1. Data is presented as mean ± SEM (*n* = 8). * represent significant (*p* < 0.05) differences between groups.
